# Effect of a Low-Carbohydrate Diet With or Without Exercise on Anxiety and Eating Behavior and Associated Changes in Cardiometabolic Health in Overweight Young Women

**DOI:** 10.3389/fnut.2022.894916

**Published:** 2022-07-06

**Authors:** Mingzhu Hu, Qingde Shi, Shengyan Sun, Hin Ieong Hong, Haifeng Zhang, Fengxue Qi, Liye Zou, Jinlei Nie

**Affiliations:** ^1^Faculty of Education, University of Macau, Macao, Macao SAR, China; ^2^Faculty of Health Sciences and Sports, Macao Polytechnic University, Macao, Macao SAR, China; ^3^Institute of Physical Education, Huzhou University, Huzhou, China; ^4^Chan Sui Ki Perpetual Help College, Macao, Macao SAR, China; ^5^College of Physical Education, Hebei Normal University, Shijiazhuang, China; ^6^Sports, Exercise and Brain Sciences Laboratory, Beijing Sport University, Beijing, China; ^7^Exercise Psychophysiology Laboratory, Institute of KEEP Collaborative Innovation, School of Psychology, Shenzhen University, Shenzhen, China

**Keywords:** low-carbohydrate diet, exercise, anxiety, eating behavior, cardiometabolic health

## Abstract

**Background:**

The effectiveness of low-carbohydrate diets (LCDs) on weight loss and exercise for improving cardiometabolic fitness have been well documented in the literature, but the effects of LCDs and whether adding exercise to a LCD regime could additionally benefit mental health (e. g., by lowering the level of anxiety) and associated changes in eating behavior are less clear in overweight and obese populations. Therefore, this study aimed to investigate the effects of a 4-week LCD with or without exercise on anxiety and eating behavior, and to explore the associations between changes in the psychological state and physiological parameters (i.e., body composition, aerobic fitness, blood pressure, lipid profile, and metabolic hormones).

**Methods:**

Seventy-four overweight Chinese women [age: 20.8 ± 3.0 years, body mass index (BMI): 25.3 ± 3.3 kg·m^−2^] completed the 4-week randomized controlled trial, which included a LCD group (i.e., ~50 g daily carbohydrate intake) with exercise training 5 days/week (LC-EXE, *n* = 26), a LCD group without exercise training (LC-CON, *n* = 25) and a control group that did not modify their habitual diets and physical activity (CON, *n* = 23). Levels of anxiety, eating behavior scores and physiological parameters (i.e., body weight, V̇O_2peak_, blood pressure, fasting glucose, blood lipids, and serum metabolic hormones including insulin, C-peptide, leptin, and ghrelin) were measured before and after the intervention.

**Results:**

There were significant reductions in anxiety levels in the LC-EXE compared with the LC-CON group, while no statistical changes were found in eating behaviors in any conditions after the 4-week intervention. Significant reduction in weight (~3.0 kg or 4%, *p* < 0.01) and decreases in insulin (~30% *p* < 0.01), C-peptide (~20% *p* < 0.01), and leptin (~40%, *p* < 0.01) were found in both LC-CON and LC-EXE groups, but adding exercise to a LCD regime generated no additional effects. There were significant improvements in V̇O_2peak_ (~15% *p* < 0.01) and anxiety (~25% *p* < 0.01) in the LC-EXE compared with the LC-CON group, while no statistical differences were found between CON and LC-CON treatments. Further analysis revealed a negative association (*r* = −0.32, *p* < 0.01) between changes in levels of anxiety and changes in V̇O_2peak_ in all participates, no other correlations were found between changes in psychological and physiological parameters.

**Conclusion:**

Although the combination of a LCD and exercise may not induce additional reductions in body weight in overweight young females, exercise could be a useful add-on treatment along with a LCD to improve cardiometabolic health and lower anxiety levels.

## Introduction

Overweight and obese individuals suffer from aesthetic concerns; more importantly, they are at a higher risk of numerous health problems, including cardiovascular diseases, type 2 diabetes, and other comorbidities (1, 2). Low-carbohydrate diets (LCDs) have been popularized in recent years for weight loss, and a growing number of studies have proven the effectiveness of this nutritional approach of restricting carbohydrate intake to induce rapid and palpable weight reduction (3–6). By limiting carbohydrate intake to <50 g or 10% of total energy intake per day and increasing fat or protein intake, LCDs mimic the body's metabolic adaption called ketosis during fasting with an increase in fat oxidation and a reduction in lipogenesis (7). Reducing dietary carbohydrates during LCDs theoretically leads to decreased blood glucose concentrations, which explains the reason why LCDs have also been advocated in the treatment of type 2 diabetes (8–10) in addition to weight reduction.

The association between dietary modifications and variations in mood and behavior has emerged as an additional area of research interest over the years (11, 12). However, the psychological effects of LCDs on anxiety levels and eating behavior have received less attention than their physiological impacts. There is some evidence suggesting that LCDs could improve psychological outcomes such as mood, depression and anxiety or could even be used as an anxiolytic approach (13), yet compared to other weight-loss diets, LCDs showed similar effects on these outcomes (14). Quite the contrary, some argues that LCDs could cause adverse effects on mood, because limited carbohydrate intake could lead to decreased glucose availability, which is an essential energy source for the brain to generate serotonin and other biochemical mood regulators (15). More importantly, the intricate relationships among modifications of nutrition intake, changes in psychological states and physiological changes, such as weight loss, changes in hormone levels (e.g., decreased levels of leptin and increased levels of ghrelin after LCDs) and cardiometabolic health (e.g., blood pressure, blood lipids), bring about difficulties in elucidating how LCDs with extremely low carbohydrate intake would impact anxiety levels or eating behavior. Although individual associations were found in previous studies between blood lipid levels (16), blood pressure (17), or metabolic hormones (e.g., leptin) (18), and levels of anxiety, it is still unknown whether changes in any of these biomarkers relate to changes in psychological status after LCDs.

Switching to LCDs from diets with relatively high carbohydrate contents and the potential impact on the psychological state during LCDs could lead to variance in eating behaviors, though little research has been designed to investigate this issue. Changes in eating behaviors should be taken into consideration when evaluating a dietary program, as body weight maintenance or weight regain could be largely affected by emotional or environmental stimuli and psychological restraint in increasing food intake (19). Notably, two recent reports suggested positive effects of LCDs on eating behavior. One uncontrolled intervention study demonstrated that a 12-week LC resulted in decreased emotional and external eating in obese adults (20), and dietary restraint was also increased significantly according to the report from another non-randomized controlled study (21). However, more studies with larger sample sizes and randomized controlled designs are needed to support the beneficial effects of LCDs on eating behaviors.

In addition to dietary approaches, alteration of sedentary behavior is also crucial for overweight individuals to prevent weight gain and improve overall health. It is well-proven in the literature that increasing one's physical activity level (i.e., moderate-intensity continuous training, MICT) is linked to better cardiorespiratory fitness, improved psychological well-being and reduced cardiovascular risk (22, 23). Therefore, the combination of exercise and a LCD could be used as a more effective lifestyle therapy to induce favorable outcomes in health conditions for overweight subjects. Different from the traditional MICT, high-intensity interval training (HIIT) or sprint interval training with a shorter work duration has generated interest in recent years due to its higher time-efficiency (24, 25). Our previous studies investigated the effects of 4-week interventions of MICT and HIIT in combination with LCDs in overweight young women, and the results suggested that the LCD intervention alone induced marked beneficial physiological effects, while the combination of exercise and LCDs additionally improved cardiorespiratory fitness (26, 27). However, it is still unknown whether LCDs, alone or in combination with MICT or HIIT, could result in a different psychological impact on levels of anxiety and eating behavior. More importantly, the interrelationship between physiological and psychological changes after a LCD and exercise intervention would be worth analyzing to explicitly understand the effects of LCDs and the potential add-on effects of exercise on these changes.

Therefore, the primary aim of the current study was to evaluate the psychological effects of a 4-week LCD intervention with *ad libitum* energy intake but restriction in carbohydrate intake (i.e., 10% of total energy intake per day), alone or in combination with exercise (i.e., MICT or HIIT), and to investigate the correlations between changes in psychological parameters and physiological indexes. Psychological outcomes included changes in anxiety levels and eating behavior, while physiological outcomes included changes in body mass, glucose profiles, lipid profiles, blood pressure, metabolic hormones, including ghrelin and leptin, and cardiorespiratory fitness indicated by V̇O_2peak_ after the intervention. It was hypothesized that the short-term LCD intervention alone would alleviate anxiety and improves eating behavior, while additional exercise training would induce additional improvements in these parameters.

## Methods

### Participants

This parallel-group, randomized controlled trial was conducted at the kinesiology laboratory at the University of Macau after obtaining the approval from the Research Ethics Panel of the University of Macau (RC Ref. no. MYRG2017-00199-FED). Based on a moderate effect size of 0.41 reported in a previous study (28), a sample of 20 in an exercise group would be adequate to distinguish differences in anxiety symptoms in a two-way repeated-measures ANOVA (two groups and two repeated measures) with a power of 80% and a significance level of 5%. To compensate for an anticipated dropout rate of 25%, the sample size was inflated to 27 participants per group.

A total of 81 Chinese female participants who completed a medical history check and the physical activity readiness questionnaire (PAR-Q), and met the following inclusion criteria were enrolled: (1) healthy young adults with no smoking or drinking habits aged between 18 and 30 years; (2) overweight or obese with a body mass index (BMI) ≥ 23 kg·m^−2^ according to the cut-off point for the Asian population (29); and (3) physically inactive, defined as no participation in regular exercise or any structured exercise for at least 6 months before enrolment. Participants were excluded if they (1) had been diagnosed with cardiovascular or metabolic disease or any other conditions that could influence physical activity and diet, or been diagnosed with any mental conditions (e.g., severe anxiety) and regularly taking medications; (2) were smokers or alcoholics or had quit smoking or drinking in the past 6 months; (3) had experienced unstable body weight over the past 6 months or were following any special dietary regimen; or (4) had been physically active over the past 6 months. Participants provided written informed consent and were informed of the detailed study procedure prior to random assignment into one of the following three groups (27 participants in each group): (1) a low-carbohydrate diet group (LC-CON); (2) a low-carbohydrate diet and exercise group (LC-EXE) and (3) a control group with no dietary or exercise intervention (CON). Allocations were concealed from the participants and the research group until participants were assigned to interventions. Four participants in the CON group, two participants in the LC-CON group and one participant in the LC-EXE were not included in the data analysis due to failure in complying with the diet (*n* = 3) and dropping out (*n* = 4) because of personal reasons during the first week of intervention. Therefore, a total of 74 participants (25 in LC-CON, 26 in LC-EXE and 23 in CON) who completed the intervention and assessments were included in the data analysis.

### Study Materials and Design

The experimental procedure consisted of a preliminary stage, pre-intervention assessments, a 4-week intervention, and post-intervention assessments. All subjects were requested to refrain from any extra physical activity other than habitual daily activity and any change of habitual diet apart from the 4-week LCD intervention.

#### Preliminary Stage

After enrolment, participants were instructed to record their daily activities and food intakes 3 days a week (2 week days and 1 weekend day) for 2 weeks before the intervention. Participants in the LC-CON and LC-EXE attended nutrition workshops given by a dietitian to receive detailed dietary instructions on the LCD and learn the methods of measuring and recording the weight and amount of food intake using standard food measuring utensils as well as the use of urine ketone tests. The menstrual cycles of all participants over the past 3 months were recorded to estimate their individual menstrual cycle. The pre- and post-intervention measurements were taken at the same menstrual stage for each participant.

#### Pre- and Post-intervention Assessment

##### Blood Sampling

Fasting blood sampling for fasting glucose and lipid profiles including total cholesterol (CHOL), low-density lipoprotein cholesterol (LDL-C), high-density lipoprotein cholesterol (HDL-C) and total triglycerides (TG) as well as metabolic regulatory hormones including insulin, C-peptide, glucagon, leptin and ghrelin were performed 72 hours before the 4-week intervention as the baseline and 72 hours after the intervention. In addition to fasting for 12 hours overnight, the subjects were required to avoid consuming caffeine or alcohol for 48 hours prior to blood sampling. The subjects arrived at the laboratory before 9 a.m. on the testing day, and then a registered nurse extracted 5 mL blood samples from their cubital veins using serum separation tubes. The blood was allowed to clot for 1 hour at room temperature and was then centrifuged at 3,000 rpm for 10 min and then frozen immediately at −80°C for later analysis. All blood samples were assayed using an automatic biochemical analyser (Olympus AU400, Olympus, Tokyo, Japan) using standard procedures in accordance with the manufacturer's instructions (KingMed Diagnostics Co., Ltd., Guangzhou, China). The intra-assay and inter-assay coefficients of variation were all below 3%.

##### Blood Pressure and Anthropometric Assessments

Assessments of blood pressure and anthropometric parameters were conducted after blood sampling. Blood pressure was measured twice on the right arm of each participant using an electronic sphygmomanometer (Microlife 3BTO-A, Taipei, Taiwan) after a 10-min seated rest. If the discrepancy between the two values of blood pressure was larger than 5 mmHg, a third measurement was performed, and the two nearest values were averaged and recorded as systolic blood pressure (SBP) and diastolic blood pressure (DBP). The mean arterial pressure (MAP) was calculated as follows: (SBP + 2 × DBP)/3.

Body mass index (BMI) in kg·m^−2^ was calculated from weight (kg) and height (m), measured using standard methods (i.e., in light clothing and without shoes). Waist circumference (WC) was measured halfway between the lower ribs and the iliac crest at the end of a normal expiration with a relaxed abdomen. Hip circumference was measured at the point of largest circumference around the buttocks. Both measurements were performed with the subject wearing minimal clothing, and the WC and HC values were recorded to the nearest 0.5 cm. The waist-to-hip ratio (WHR) was calculated as the ratio between WC and HC.

##### Maximal Incremental Exercise Test

Maximal incremental exercise tests were carried out before and 72 h after the intervention to calculate each participant's cardiorespiratory fitness (e.g., V̇O_2peak_). The tests were performed on a computer-controlled cycle ergometer (Monark 839E, Varberg, Sweden). Participants warmed up on the cycle ergometer at a workload of 25 W for 3 min and started cycling at 50 W, which increased by 25 W every 3 min. Participants maintained their cycling cadence at 60 ± 5 rpm until exhaustion, which was assessed by the following criteria: (1) rest exchange ratio (RER) ≥ 1.15; (2) ratings of perceived exertion (RPE) ≥ 18; (3) HR_peak_ ≥ 90% of age-predicted maximum (220-age) (30). Respiratory gases were acquired continuously by the Vmax Encore System (CareFusion Corp., San Diego, CA, USA) throughout the test, and V̇O_2peak_ was defined as the highest oxygen consumption value averaged over 15 s of the final stage.

##### Assessment of Eating Behavior

The Dutch Eating Behavior Questionnaire (DEBQ) (31) was employed to assess eating behavior before and after the 4-week intervention. The DEBQ consists of 33 individual items assessing three domains of eating behavior including restrained eating (10 items), emotional eating (13 items) and external eating (10 items). Sample items in the DEBQ would be “do you try to eat less at mealtimes than you would like to eat?” for questions about restrained eating, “do you have a desire to eat when you are irritated?” for emotional eating and “do you eat more than usual if food tastes good to you?” for external eating. Each item was scored on a scale ranging from 1 (never) to 5 (very often). The DEBQ showed good reliability and validity for assessing eating behavior, with alpha coefficients ranging from 0.92 to 0.94 for the restrained eating subscale, 0.96 to 0.97 for the emotional eating subscale and 0.79 to 0.84 for the external eating subscale (31).

##### Assessment of Anxiety

The 7-item Generalized Anxiety Disorder Scale (GAD-7) was used to assess levels of anxiety or identify probable cases of generalized anxiety disorder (GAD), before and after the 4-week intervention. Seven core symptoms of GAD were identified by frequency of occurrence during the last 2 weeks. Sample items on the scale would be “feeling nervous, anxious or on edge”, “not being able to stop or control worrying” or “feeling afraid as if something awful might happen”. Subjects responded to each item on a scale from 0 (not at all) to 3 (nearly every day). The total possible score on the GAD-7 ranges from 0 to 21, with scores of ≥5, ≥10 and ≥15 representing mild, moderate, and severe anxiety levels, respectively. The GAD-7 has been shown to be a reliable and valid brief self-reported scale (Cronbach's alpha = 0.92 and intraclass correlation = 0.83) (32) not only in clinical practice but also as a measure of anxiety in the general population (33).

#### Dietary Intervention

Participants in the LC-CON and LC-EXE consumed a LCD with macronutrients consisting of approximately 65% fats, 25% proteins, and 10% carbohydrates (~50 g/day) for 4 weeks. Participants learned how to choose appropriate foods for the LCD during the preliminary stage and were provided with personal dietary guidance and support from the dietitian during the 4-week intervention. A handbook listing sample foods/drinks and recipes for LCDs were given to the participants. The appropriate foods for a LCD include all kinds of fat from saturated or unsaturated sources, meats including all kinds of beef, fish, chicken, duck, pork, seafoods, non-starchy vegetables, cheese, eggs, nuts/seeds, oils, low-carbohydrate drinks such as black coffee, green tea or black tea, and water. Participants were also encouraged to add five tablespoons of olive oil daily. All high-carbohydrate-containing foods, such as rice, noodles, sweets and alcoholic beverages, were prohibited during the intervention.

Adherence to the LCD was assessed by daily urinary ketone tests and validated food diaries 3 days a week (2 week days and 1 weekend day) during the intervention. Daily urinary ketone tests were performed, and their results were recorded by the participants themselves every morning or after dinner using reagent strips (UROPAPER, Suzhou First Pharmaceutical Co. Ltd., Suzhou, China). Records of the test and food diaries were reported to the researchers weekly. Compliance with LCDs was verified by urine ketones detected as a color change on the reagent strip, which is 95.1% concordant with clinical diagnosis (34). Food records were analyzed by a dietitian using the nutrition analysis and management software (NRISM, version 3.1, China), which also acted as a verification of dietary compliance. Participants received personalized dietary guidance and support from the dietitian based on the records and analysis.

#### Exercise Intervention

All subjects were required to maintain their habitual physical activity levels, which were assessed daily using validated pedometers (Yamax Digi-Walker SW-200, Tokyo, Japan), while subjects in the LC-EXE group participated in additional exercise training 5 days/week at the kinesiology laboratory, with strictly controlled room temperature (22°C) and humidity (50–60%), under the supervision of two trained research assistants. Subjects in the LC-EXE group were randomly allocated to a MICT training program (*n* = 13) or a HIIT training program (*n* = 13). The MICT training consisted of 30 min of continuous cycling with a speed of 50 ± 5 rpm at 50% of V̇O_2peak_ for the first 10 training sessions and 60% of V̇O_2peak_ for the last 10 training sessions on a Monark cycle ergometer (839E, Varberg, Sweden). The HIIT training consisted of 10 repetitions of 6-s “all-out” cycling bursts interspersed with 9-s passive recoveries on the Monark cycle ergometer (839E, Varberg, Sweden). The workload during sprint cycling was initially 1 kg and increased by 0.5 kg once the participants were able to maintain a cycling speed of more than 100 rpm for all sprint bouts in two consecutive training sessions until the workload reached 5% of their own body weight. During MICT training, RPE, (Borg 6–20 scale) (32) and heart rate (HR) measured by a HR monitor (Polar F4M BLK, Kempele, Finland) were recorded every 5 min. During HIIT, RPE was recorded immediately before the 1st sprint bout and immediately after the 5th and 10th sprint bouts, and HR was recorded immediately before and immediately after each sprint bout. The power output every 5 min during MICT and for each exercise bout in HIIT were recorded automatically by the Monark Anaerobic Test software.

#### Statistical Analysis

The PASW software (Release 27.0; IBM, New York, NY, USA) was used to perform statistical analyses. Prior to the main statistical analyses, Shapiro–Wilk tests were performed to confirm whether the outcome variables were normally distributed. Analysis of covariance (ANCOVA) with pre-intervention values as the covariate was used to examine the group differences in the main outcome variables among the three groups. One-way ANOVA tests were computed to assess group differences in changes in outcome variables after the intervention and the daily activities, energy intake and macronutrient proportions. Effect sizes are presented as partial eta-squared (partial η^2^) among the three groups and Cohen's d values for the difference between variables. The Pearson product-moment correlation coefficient test was used to examine the association between psychological parameters and physiological changes. Data are presented as mean ± standard deviation, and the significance level was set at *p* < 0.05.

## Results

### Dietary Composition and Diet Compliance

At baseline, the three groups had similar daily dietary energy intakes (~2.040 kcal), corresponding to approximately 45% carbohydrate, 15% protein, and 35% fat. During the 4-week intervention, participants in LC-CON and LC-EXE groups adhered to the prescribed LCD, which had a macronutrient composition of 65% fats, 25% proteins, and 10% carbohydrates, with no change in energy intake from baseline (Supplementary Table 1). Moreover, participants were positive for urinary ketosis on 96.7 ± 5.9% and 96.0 ± 7.9% of the days in the LC-CON and LC-EXE, respectively, after 3 days of LCD consumption, indicating good adherence to the LCD.

### Daily Physical Activity and Training Data

Daily physical activity assessed by the pedometer were not differ between groups (Supplementary Table 1). In the LC-EXE group, participants performed 20 additional sessions of HIIT or MICT (i.e., 5 sessions a week for 4 weeks). Training times were 12.5 min weekly and 50 min in total (4 weeks) for HIIT, and 150 min weekly and 600 min in total for MICT. During the exercise sessions, HIIT and MICT elicited an average HR of 147 ± 11 bpm and 144 ± 6 bpm, respectively, yet the RPE during HIIT was 13 ± 2, which was higher than that in MICT (11 ± 1, *p* < 0.01).

### Anthropometric Parameters, Blood Pressure and Cardiorespiratory Fitness

Outcomes for anthropometric parameters, blood pressure and V̇O_2peak_ before and after the 4-week intervention are presented in Table 1. No statistical differences in any of the outcomes were found across the three groups at baseline. After intervention, the LC-CON and LC-EXE groups had significant reductions in weight and BMI by ~4%, and HC by ~2.5% on post-intervention measurements (Tables 1, 2). Among the three groups, only LC-EXE resulted in significantly reduced SBP (−5 ± 8 mmHg), MAP (−3 ± 6 mmHg) and increased V̇O_2peak_ (15.9 ± 11.4%), while no statistical differences were found in SPB, MAP or V̇O_2peak_ in the LC-CON before and after the intervention. No significant changes were found in DBP in LC-CON nor LC-EXE. Importantly, there were significant improvements in V̇O_2peak_ in LC-EXE compared to LC-CON after the intervention, although the two groups had similar baseline V̇O_2peak._

**Table 1:** Anthropometric and physiological outcomes and blood pressure before and after 4 weeks of intervention.

	**CON (*****n*** **=** **23)**		**LC-CON (*****n*** **=** **25)**		**LC-EXE (*****n*** **=** **26)**		**Group effect**	
	**Pre**	**Post**	**Pre**	**Post**	**Pre**	**Post**	***p***	***Partial η^2^***
Age (y)	20.3 ± 2.3		20.5 ± 3.5		21.6 ± 3.1		0.290	
Height (cm)	162.6 ± 5.3		162.4 ± 4.1		161.8 ± 5.9		0.849	
Weight (kg)	66.5 ± 9.4	66.3 ± 9.9	66.8 ± 10.5	64.4 ± 9.9^*^	66.2 ± 8.2	63.2 ± 8.1^*^	<0.001	0.376
BMI (kg·m^−2^)	25.1 ± 3.1	25.0 ± 3.2	25.5 ± 3.8	24.4 ± 3.8^*^	25.3 ± 2.7	24.2 ± 2.8^*^	<0.001	0.399
WC (cm)	77.1 ± 8.6	73.8 ± 8.1	79.1 ± 9.9	75.6 ± 9.1	77.2 ± 6.9	72.9 ± 6.1	<0.001	0.211
HC (cm)	99.6 ± 6.2	98.9 ± 6.8	102.2 ± 7.2	99.3 ± 6.9^*^	101.6 ± 5.3	99.2 ± 5.0^*^	<0.001	0.261
WHR	0.77 ± 0.05	0.78 ± 0.05	0.78 ± 0.05	0.76 ± 0.05^†^	0.77 ± 0.05	0.74 ± 0.04^†^	0.003	0.154
SBP (mmHg)	111 ± 11	113 ± 12	111 ± 13	109 ± 11	112 ± 9	107 ± 11^*^	0.039	0.088
DBP (mmHg)	72 ± 10	70 ± 9	72 ± 10	70 ± 9	71 ± 5	68 ± 7	0.061	0.077
MAP (mmHg)	85 ± 10	86 ± 9	85 ± 10	83 ± 10	84 ± 6	81 ± 8^*^	0.031	0.095
V̇O_2peak_ (ml·min^−1^·kg^−1^)	25.4 ± 3.8	25.3 ± 4.7	25.4 ± 4.5	24.4 ± 3.7	23.5 ± 3.6	27.0 ± 3.7^*#^	<0.001	0.284

*CON, control group; LC-CON, low-carbohydrate diet control group; LC-EXE, low-carbohydrate diet combined with exercise training (high-intensity interval training or moderate-intensity continuous training); BMI, body mass index; WC, waist circumference; HC, hip circumference; WHR, waist-to-hip ratio; SBP, systolic blood pressure; DBP, diastolic blood pressure; MAP, mean arterial pressure; V̇O_2peak_, peak oxygen uptake*.

*Significant difference from CON at ^†^p < 0.01*,

* *p < 0.01*,

*significant difference from LC-CON at ^#^p < 0.01*.

**Table 2:** Changes in outcome variables after intervention.

	**CON**	**LC-CON**	**LC-EXE**	**ES** ***(d)***	
	**(*n* = 23)**	**(*n* = 25)**	**(*n* = 26)**	**LC-CON**	**LC-EXE**
Δ Weight (kg)	−0.27 ± 1.61	−2.38 ± 1.82^**^	−2.97 ± 1.08^**^	1.13	1.99
Δ BMI (kg·m^−2^)	−0.11 ± 0.61	−0.91 ± 0.68^**^	−1.13 ± 0.39^**^	1.14	2.02
Δ WC (cm)	−3.26 ± 2.68	−3.52 ± 2.69	−4.31 ± 2.44	0.10	0.41
Δ HC (cm)	−0.70 ± 1.71	−2.94 ± 2.54^**^	−2.45 ± 1.73^**^	0.86	1.02
Δ WHR	0.01 ± 0.02	−0.02 ± 0.02^**^	−0.02 ± 0.02^**^	0.97	1.70
Δ SBP (mmHg)	2 ± 9	−2 ± 10	−5 ± 8^*^	0.40	0.79
Δ DBP (mmHg)	1 ± 6	−2 ± 7	−2 ± 5	0.49	0.57
Δ MAP (mmHg)	1 ± 6	−2 ± 7	−3 ± 6^*^	0.50	0.76
Δ V̇O_2peak_ (ml·min^−1^·kg^−1^)	−0.11 ± 3.76	−1.01 ± 3.59	3.56 ± 2.42^**^^#^	0.25	1.18
Δ V̇O_2peak_%	−0.07 ± 13.99	−1.36 ± 12.3	15.9 ± 11.4^**^^#^	0.10	1.26
Δ FG (mmol·L^−1^)	0.18 ± 0.39	0.01 ± 0.44	−0.15 ± 0.37^*^	0.38	0.88
Δ CHOL (mmol·L^−1^)	0.11 ± 0.62	0.84 ± 1.08^*^	0.73 ± 0.72^*^	0.66	0.91
Δ HDL-C (mmol·L^−1^)	0.00 ± 0.16	0.09 ± 0.26	0.11 ± 0.17	0.33	0.65
Δ LDL-C (mmol·L^−1^)	0.13 ± 0.55	0.76 ± 0.95^*^	0.71 ± 0.63^*^	0.65	0.98
Δ TG (mmol·L^−1^)	−0.06 ± 0.29	−0.01 ± 0.61	−0.20 ± 0.38	0.09	0.38
Δ Insulin (μIU·ml^−1^)	−0.9 ± 7.0	−4.5 ± 5.8^**^	−3.8 ± 3.1^**^	0.83	0.90
Δ C-peptide (pg·ml^−1^)	25.3 ± 358.5	−193.3 ± 374.2	−257.5 ± 483.6^*^	0.60	0.66
Δ Leptin (ng·ml^−1^)	−1.5 ± 7.6	−4.3 ± 6.8	−6.3 ± 7.9^*^	0.39	0.62
Δ Ghrelin (pg·ml^−1^)	−17.2 ± 393.1	−19.6 ± 373.8	−100.3 ± 195.3	0.01	0.28

*Delta (Δ), change from pre- to post-intervention; CON, control group; LC-CON, low-carbohydrate diet control group; LC-EXE, low-carbohydrate diet combined with exercise training (high-intensity interval training or moderate-intensity continuous training); BMI, body mass index; WC, waist circumference; HC, hip circumference; WHR, waist-to-hip ratio; V̇O_2_peak, peak oxygen uptake; FG, fasting glucose; CHOL, total cholesterol; HDL-C, high-intensity lipoprotein cholesterol; LDL-C, low-intensity lipoprotein cholesterol; TG, triglycerides*.

Significant difference from CON at ^*^p < 0.05, ^**^p < 0.01,

*significant difference from LC-CON at ^#^p < 0.01. Cohen's d value for effect size (ES) when compared to the CON group*.

### Blood Parameter Outcomes

The results of blood parameter outcomes including fasting glucose, blood lipid profiles and several metabolic regulatory hormones and changes in these outcomes from pre- to post-intervention are presented in Tables 2, 3. At baseline, there were no significant differences in any of the outcomes across the three groups. After the intervention, no statistical changes were found in fasting glucose across the three groups. For blood lipid profiles, both LC-CON and LC-EXE resulted in significantly higher CHOL (16.5% in LC-CON and 16.1% in LC-EXE) and LDL-C (23.9% in LC-CON and 25.5% in LC-EXE) on post-intervention measurements after the intervention, despite while no differences were found between the two groups. HDL-C and TG were not statistically changed after the intervention in all groups. For post-intervention measurements of metabolic regulatory hormones, there were significant reductions in insulin (-31.9% in LC-CON and −24.2% in LC-EXE), C-peptide (−17.1% in LC-CON and −24.8% in LC-EXE) and leptin in LC-CON and LC-EXE (−32.7% in LC-CON and −53.5% in LC-EXE) after the intervention, with no significant differences between the two groups. No statistical changes were observed for ghrelin in all the three groups.

**Table 3:** Blood parameter outcomes before and after 4 weeks of intervention.

	**CON (*****n*** **=** **23)**		**LC-CON (*****n*** **=** **25)**		**LC-EXE (*****n*** **=** **26)**		**Group effect**	
	**Pre**	**Post**	**Pre**	**Post**	**Pre**	**Post**	***p***	***Partial η^2^***
FG (mmol·L^−1^)	4.5 ± 0.5	4.7 ± 0.4	4.7 ± 0.4	4.7 ± 0.4	4.9 ± 0.4	4.7 ± 0.5	0.247	0.041
CHOL (mmol·L^−1^)	4.8 ± 1.1	4.9 ± 1.0	5.1 ± 1.0	5.9 ± 1.4^*^	4.5 ± 0.6	5.2 ± 1.0^*^	0.007	0.140
HDL-C (mmol·L^−1^)	1.6 ± 0.4	1.6 ± 0.4	1.6 ± 0.4	1.6 ± 0.4	1.5 ± 0.3	1.6 ± 0.3	0.204	0.047
LDL-C (mmol·L^−1^)	3.0 ± 1.0	3.1 ± 0.9	3.2 ± 0.9	4.0 ±1.3^*^	2.8 ± 0.5	3.5 ± 0.8^*^	0.005	0.149
TG (mmol·L^−1^)	1.1 ± 0.8	1.1 ± 0.9	1.1 ± 0.9	1.1 ± 0.8	1.1 ± 0.4	0.9 ± 0.2	0.216	0.045
Insulin (μIU·ml^−1^)	14.8 ± 10.7	15.7 ± 8.6	15.8 ± 8.4	11.1 ± 8.4^*^	14.0 ± 7.8	10.2 ± 6.1^*^	<0.001	0.208
C-peptide (pg· ml^−1^)	1,125.5 ± 424.9	1,150.9 ± 384.8	1,038.0 ± 506.1	844.8 ± 428.1^*^	1,133.3 ± 423.1	875.7 ± 340.6^*^	0.007	0.136
Leptin (ng· ml^−1^)	14.1 ± 9.6	12.6 ± 6.9	11.8 ± 7.6	7.5 ± 5.9^*^	13.2 ± 6.5	6.9 ± 5.0^*^	0.002	0.168
Ghrelin (pg· ml^−1^)	839.1 ± 682.1	821.9 ± 583.3	747.3 ± 584.6	727.7 ± 633.1	673.4 ± 727.7	573.2 ± 346.6	0.370	0.031

*CON, control group; LC-CON, low-carbohydrate diet control group; LC-EXE, low-carbohydrate diet combined with exercise training (high-intensity interval training or moderate-intensity continuous training); FG, fasting glucose; CHOL, total cholesterol; HDL-C, high-intensity lipoprotein cholesterol; LDL-C, low-intensity lipoprotein cholesterol; TG, triglycerides*.

Significant difference from CON at ^*^p < 0.01.

### Anxiety Level and Eating Behavior

As presented in Table 4, the three groups started with mild anxiety levels before the intervention, with no significant differences across groups, yet only LC-EXE resulted in a significant decrease in anxiety levels (−26.9%) after the 4-week intervention. No statistical changes on post-intervention measurements in anxiety were found in CON or LC-CON despite increases in ~20% in CON and ~40% in LC-CON.

**Table 4:** Outcome variables before and after 4 weeks of intervention.

	**CON (*****n*** **=** **23)**		**LC-CON (*****n*** **=** **25)**		**LC-EXE (*****n*** **=** **26)**		**Group effect (** ***time*** **)**	
	**Pre**	**Post**	**Pre**	**Post**	**Pre**	**Post**	***p***	***Partial η^2^***
GAD-7	5.1 ± 3.6	6.1 ± 3.8	5.0 ± 3.6	7.4 ± 5.1	6.1 ± 2.5	4.7 ± 3.1^*^^#^	0.001	0.173
DEBQ-a	28.9 ± 5.7	29.6 ± 4.8	29.6 ± 6.4	32.9 ± 6.7	28.1 ± 6.3	31.1 ± 7.4	0.154	0.052
DEBQ-b	38.4 ± 11.5	40.9 ± 13.2	38.1 ± 11.4	38.0 ± 11.4	33.2 ± 10.5	33.6 ± 10.9	0.332	0.031
DEBQ-c	34.6 ± 5.3	33.5 ± 4.2	34.8 ± 6.1	33.4 ± 5.9	32.6 ± 3.6	32.2 ± 5.7	0.982	0.001

*CON, control group; LC-CON, low-carbohydrate diet control group; LC-EXE, low-carbohydrate diet combined with exercise training (high-intensity interval training or moderate-intensity continuous training); GAD-7, general anxiety disorder 7-item; DEBQ-a, domain of restrained eating of the Dutch Eating Behavior Questionnaire; DEBQ-b, domain of emotional eating of the Dutch Eating Behavior Questionnaire; DEBQ-c, domain of external eating of the Dutch Eating Behavior Questionnaire*.

Significant difference from CON at ^*^p < 0.05,

*significant difference from LC-CON at ^#^p < 0.01*.

No statistical changes were found in any of the three subscales of the DEBQ (i.e., restricted eating presented in DEBQ-a, emotional eating presented in DEBQ-b and external eating presented in DEBQ-c) after the intervention in the three groups.

### Correlations

Correlations between changes in outcomes after the intervention are presented in Table 5. Changes in anxiety level, indicated by scores on the GAD-7, were positively correlated with changes in emotional eating behavior (DEBQ-b) (i.e., a higher anxiety level was associated with more prominent emotional eating) (*r* = 0.23, *p* < 0.05). Moreover, changes in anxiety level were negatively correlated with changes in V̇O_2peak_ (*r* =−0.32, *p* < 0.01), indicating that lower anxiety levels were associated with greater improvements in V̇O_2peak_. Correlations between all the other outcomes were not statistically significant.

**Table 5:** Relationships between physiological and psychological parameters.

	**Δ GAD-7**	**Δ DEBQ-a**	**Δ DEBQ-b**	**Δ DEBQ-c**
Δ Weight (kg)	0.26^*^	−0.10	0.17	−0.04
Δ BMI (kg·m^−2^)	0.26^*^	−0.10	0.17	−0.04
Δ V̇O_2peak_	−0.32^**^	0.14	−0.07	0.02
Δ HC (cm)	0.27^*^	0.11	0.08	0.04
Δ FG (mmol·L^−1^)	0.23 (*p* = 0.058)	−0.24^*^	−0.02	−0.13
Δ CHOL (mmol·L^−1^)	−0.06	0.05	0.07	−0.04
Δ HDL-C (mmol·L^−1^)	−0.06	0.00	0.12	−0.07
Δ LDL-C (mmol·L^−1^)	−0.10	0.02	0.06	−0.07
Δ TG (mmol·L^−1^)	0.19	0.22	0.04	0.11
ΔInsulin (μIU·ml^−1^)	0.08	0.08	0.21	0.21
ΔC-peptide (pg·ml^−1^)	0.07	−0.16	0.06	−0.03
ΔLeptin (ng·ml^−1^)	0.10	−0.14	0.11	−0.04
ΔGhrelin (pg·ml^−1^)	−0.08	−0.10	0.03	0.05
Δ GAD-7	–	−0.12	0.23^*^	−0.07

*Delta (Δ), change from pre- to post-intervention; GAD-7, general anxiety disorder 7-item; DEBQ-a, domain of restrained eating of the Dutch Eating Behavior Questionnaire; DEBQ-b, domain of emotional eating of the Dutch Eating Behavior Questionnaire; DEBQ-c, domain of external eating of the Dutch Eating Behavior Questionnaire; BMI, body mass index; V̇O_2_peak, peak oxygen uptake; HC, hip circumference; FG, fasting glucose; CHOL, total cholesterol; HDL-C, high-intensity lipoprotein cholesterol; LDL-C, low-intensity lipoprotein cholesterol; TG, triglycerides*.

* *p < 0.05*,

** *p < 0.01*.

## Discussion

This randomized controlled study investigated the effects of a 4-week LCD and the combined effects of a LCD and exercise on psychological outcomes including anxiety and eating behavior, and correlations between these two outcomes and the following physiological outcomes: anthropometric parameters (i.e., body weight, waist, and hip circumference), and biomarkers of cardiovascular health (i.e. blood glucose, blood lipids, blood pressure, c-peptide, insulin, ghrelin, leptin, and cardiorespiratory fitness) in overweight young females. Compared with the control group, the short-term LCD combined with an exercise intervention resulted in significant improvements in anxiety levels, while the LCD intervention alone did not induce statistical changes in anxiety. However, no significant changes in eating behavior were observed in any group. Additional exercise training also significantly decreased systolic blood pressure and improved cardiorespiratory fitness, as indicated by increased V̇O_2peak_, after the 4-week intervention. Further analysis showed a negative association between changes in V̇O_2peak_ and levels of anxiety, while no other significant correlations were found between psychological and physiological outcomes.

The state of ketosis generated by LCDs has been reported to alleviate symptoms of anxiety disorders (13), which can have a debilitating impact on individuals' daily functioning and well-being. However, contradicting our hypothesis, improvements in anxiety levels were not observed following the LCD intervention in the present study, as no significant differences between the LC-CON and CON group were observed. The value of the standard deviation of the post-intervention anxiety level in LC-CON (i.e., 5.1 or 69% of the mean) indicated potential large inter-individual differences in the experiences of short-term LCDs. In addition to the proposed reduction in serotonin concentrations during LCDs that might adversely affect anxiety levels from a physiological perspective (7), some participants reported that they encountered obstacles in their social lives that could cause them much anxiety when adhering to the LCD. Specifically, fewer food choices and doubts about their high fat intake from the people around them increased some participants' uncertainty and anxiety toward the LCD. In contrast, the participants in the LC-EXE group with extra HIIT or MICT training had significantly reduced anxiety levels compared with the LC-CON group. Exercise appears to be protective against symptoms of anxiety in people with and without anxiety disorders, according to previous reviews and meta-analyses (35–39). Our results extended this knowledge by discovering that the anti-anxiety effects of exercise could also be produced in individuals consuming LCDs. Further findings of the association between V̇O_2peak_ and anxiety levels suggested that improving cardiorespiratory fitness through exercise could be beneficial in mitigating the unfavorable effects of LCDs. Several potential mechanisms from neurobiological (e.g., endogenous opioid release), psychosocial (e.g., appearance self-perceptions) and behavioral standpoints (e.g., improved sleep quality) have been identified in the literature to explain the effects of exercise on anxiety (23). These mechanisms could also be applied to the findings of the present study that combining exercise with LCDs decreased overall anxiety levels. Despite the correlation between anxiety levels and emotional eating scores, no statistical changes were found in any subscale of eating behaviors in the LC-CON and LC-EXE groups. Conversely, significantly increased dietary restraint was reported in a 4-week study, and decreased emotional and external eating were observed in another 12-week study (20, 21). It is unknown whether differences in food cravings or food preference account for the inconsistent findings in eating behaviors, as participants with high levels of cravings for high-fat food at baseline might be satisfied with the LCD.

Consistent with previous findings that LCDs with or without calorie intake restriction efficiently reduced weight and BMI (4, 6, 40, 41), the overweight participants in the current study lost approximately 3 kg after the 4-week intervention. Moreover, the participants' hip circumferences were significantly reduced after LCDs compared to the CON, accompanied by an improved waist-to-hip ratio, suggesting a decreased risk of cardiovascular events (42, 43). However, additional effects on anthropometric parameters were not found in LC-EXE with extra exercise intervention, which is in line with our previous study (26, 27). Moreover, extra exercise did not trigger greater effects on appetite-related hormones, as both LC-CON and LC-EXE resulted in significant reductions in the anorexigenic hormone leptin but no statistical changes in the orexigenic hormone ghrelin, which could be explained largely by the moderating effects of these hormones after significant weight loss (44). Although fasting glucose was not statistically changed after LCDs regardless of with extra exercise training or not, considering that baseline values of fasting glucose were already within the healthy range for young females (i.e., <5 mmol·L^−1^), our results still confirmed the beneficial effects of LCDs on glycaemic control (8, 37, 45–47), as c-peptide and insulin concentrations were significantly lower after the intervention. Nevertheless, no significant correlations were found between reductions in weight or changes in hormones or glycaemic markers and changes in anxiety levels or eating behaviors.

It should be acknowledged that significantly higher CHOL and LDL-C but no statistical significant changes in HDL-C or triglyceride levels compared to the CON were observed after the LCD intervention, which is in line with some previous studies reporting short-term effects of LCDs on blood lipid profiles (48–50). Our novel finding is that additional exercise training did not neutralize the changes or elicit greater effects, suggesting that the effects of a short-term exercise intervention on blood lipid and lipoprotein levels might be overpowered by the impact of an altered nutritional composition of increased saturated fatty acid, CHOL and trans fatty acid intake. The major concern when adopting LCDs is that the potential detrimental effects of LCDs on LDL-C could outweigh the effects on weight loss and other health benefits, since high LDL-C concentrations are reported in association with an increased risk of cardiovascular diseases (e.g., atherosclerotic diseases) (41, 51). Nevertheless, it should be noted that, after the 4-week intervention, the LDL-C levels of the young, overweight otherwise healthy females in the current study did not reach a clinically problematic level (≥4.1 mmol/L) (52), even when considering the variations in LDL-C levels among the participants. Rises in LDL-C are often observed during short-term LCD interventions, yet findings from several meta-analyses suggested that LDL-C levels tended to return to the baseline values after a relatively long-term intervention (≥12 months) (53, 54). Moreover, significant rises in CHOL and LDL-C levels were not statistically related to changes in anxiety level, possibly due to the fact that the rises were not large enough to impact participants' general health or cause any medical problems.

Although the addition of a 4-week exercise regimen to the LCD did not produce greater effects on plasma lipid profiles than the LCD intervention alone, it resulted in markedly lowered systolic blood pressure in participants without clinical hypertension. However, changes in blood pressure did not correlate with changes in anxiety levels. In contrast to the findings of our previous study (26), the LCD alone did not lead to significant improvements in blood pressure profiles. Given that the present study and our previous study analyzed LCDs with similar macronutrient compositions, the inconsistent effects on blood pressure might be attributed to differences in micronutrients that the participants consumed during the LCD intervention. For example, sodium, potassium and the ratio between these two have been suggested to impact blood pressure levels (55, 56), and imbalanced ratios could lead to adverse blood pressure profiles. As the current LCD intervention did not tend to manipulate intakes of micronutrients, we cannot exclude the possibility that the effects of weight reduction on blood pressure might be offset by the influence of varied consumption of micronutrients. Nevertheless, further research is necessary to address this important gap in the analysis of LCDs before this assumption can be fully approved.

This study has several limitations that should be addressed. Firstly, as the present study analyzed a 4-week LCD intervention in healthy, overweight young females, it is not possible to make any conclusions, based on our findings, about the long-term effects of LCDs in other populations with varied health conditions. Secondly, we implemented both HIIT and MICT protocols in the LC-EXE group but did not perform separate analyses of these two groups due to the relatively small number of subjects in each exercise protocol, which might negate the differences between these two exercise types. Also, the analysis of psychological responses to LCDs might be limited by a lack of other related measurements (e.g., food cravings), which could be important when interpreting the effects of LCDs. Lastly, given that the participants were free to choose their preferred low-carbohydrate foods and recorded daily food intakes by themselves, micronutrient intakes during LCDs could not be analyzed in the present study, which might have an impact on physiological outcomes.

In conclusion, this study comprehensively examined the effects of a short-term LCD with or without exercise on physical and psychological health using a randomized controlled design. The 4-week LC intervention was an efficient approach to reducing body weight and improving glycaemic control in overweight young females, but caution should be exercised regarding significantly increased CHOL and LDL-C levels, as well as anxiety levels, which emphasize the necessity of professional guidance and monitoring of blood lipid profiles when adopting LCDs. Moreover, adding exercise to a low-carbohydrate dietary program could be beneficial in overcoming potential adverse effects on disturbance in anxiety levels and improving cardiorespiratory health.

## Data Availability Statement

The raw data supporting the conclusions of this article will be made available by the authors, without undue reservation.

## Ethics Statement

The studies involving human participants were reviewed and approved by the University of Macau. The patients/participants provided their written informed consent to participate in this study.

## Author Contributions

MH, SS, QS, LZ, and JN contributed to the conception and design of the manuscript. MH, SS, and HH performed data search and data extraction. MH, QS, SS, HH, HZ, and JN performed data-analysis. MH, QS, SS, HH, HZ, FQ, LZ, and JN drafted and revised the manuscript. All authors contributed to the article and approved the submitted version.

## Conflict of Interest

The authors declare that the research was conducted in the absence of any commercial or financial relationships that could be construed as a potential conflict of interest.

## Publisher's Note

All claims expressed in this article are solely those of the authors and do not necessarily represent those of their affiliated organizations, or those of the publisher, the editors and the reviewers. Any product that may be evaluated in this article, or claim that may be made by its manufacturer, is not guaranteed or endorsed by the publisher.
