# Supplementary material for: Effect of a Low-Carbohydrate Diet With or Without Exercise on Anxiety and Eating Behavior and Associated Changes in Cardiometabolic Health in Overweight Young Women
Source: Front Nutr. 2022 Jul 6;9:894916. doi: 10.3389/fnut.2022.894916 (PMC9298497; doi:10.3389/fnut.2022.894916)
Supplement: Supplementary file 1 [file Table_1.doc]

**Supplementary Table 1**. Dietary energy intake and habitual physical activity during the intervention

|  | Pre-2 week | | Pre-1 week | | Week 1 | | Week 2 | | Week 3 | | Week 4 | |
| --- | --- | --- | --- | --- | --- | --- | --- | --- | --- | --- | --- | --- |
| Energy intake (kcal∙day^-1^) | |  |  |  | 2189 | ± 503 | 2095 | ± 474 | 1994 | ± 610 | 2059 | ± 397 |
| CON |  |  |  |  | 2189 | ± 503 | 2095 | ± 474 | 1994 | ± 610 | 2059 | ± 397 |
| LC-CON | 1984 | ± 615 | 2020 | ± 339 | 1838 | ± 472 | 1671 | ± 575 | 1729 | ± 468 | 1666 | ± 464 |
| LC-EXE | 2137 | ± 389 | 2047 | ± 534 | 2194 | ± 989 | 1979 | ± 467 | 1866 | ± 435 | 1812 | ± 236 |
| Carbohydrate (%) |  |  |  |  |  |  |  |  |  |  |  |  |
| CON |  |  |  |  | 45.5 | ± 8.3 | 43.5 | ± 7.9 | 43.7 | ± 7.9 | 44.3 | ± 10.3 |
| LC-CON | 44.1 | ± 8.0 | 44.9 | ± 10.7 | 8.7 | ± 5.1 | 8.2 | ± 6.0 | 8.9 | ± 5.0 | 8.3 | ± 7.0 |
| LC-EXE | 47.1 | ± 9.0 | 47.0 | ± 9.8 | 14.5 | ± 10.0 | 12.3 | ± 5.0 | 8.7 | ± 3.2 | 7.9 | ± 2.2 |
| Fat (%) |  |  |  |  |  |  |  |  |  |  |  |  |
| CON |  |  |  |  | 38.3 | ± 6.9 | 39.9 | ± 7.3 | 40.2 | ± 6.5 | 36.6 | ± 9.6 |
| LC-CON | 35.9 | ± 7.6 | 37.5 | ± 7.3 | 68.9 | ± 7.1 | 69.5 | ± 7.1 | 69.3 | ± 5.9 | 71.1 | ± 9.0 |
| LC-EXE | 34.5 | ± 7.0 | 34.6 | ± 7.2 | 62.3 | ± 8.2 | 62.7 | ± 7.7 | 69.3 | ± 5.8 | 70.7 | ± 5.7 |
| Protein (%) |  |  |  |  |  |  |  |  |  |  |  |  |
| CON |  |  |  |  | 15.0 | ± 3.9 | 15.2 | ± 3.1 | 15.3 | ± 4.0 | 15.1 | ± 4.0 |
| LC-CON | 14.7 | ± 3.8 | 14.8 | ± 3.0 | 22.5 | ± 5.0 | 22.4 | ± 4.9 | 22.1 | ± 6.0 | 21.2 | ± 5.2 |
| LC-EXE | 13.8 | ± 2.9 | 15.0 | ± 2.9 | 23.2 | ± 5.9 | 25.1 | ± 6.2 | 22.2 | ± 5.0 | 21.6 | ± 4.7 |
| Daily steps | | |  |  |  |  |  |  |  |  |  |  |
| CON |  |  |  |  | 7862 | ± 2747 | 7992 | ± 2806 | 8464 | ± 2162 | 7704 | ± 2273 |
| LC-CON | 8442 | ± 2059 | 7646 | ± 2201 | 7405 | ± 2130 | 8106 | ± 2600 | 7871 | ± 2862 | 7427 | ± 2309 |
| LC-EXE | 8065 | ± 2872 | 7901 | ± 2179 | 8942 | ± 1791 | 8747 | ± 2340 | 8809 | ± 1678 | 8316 | ± 1973 |

CON: control group, LC-CON: low-carbohydrate diet control group, LC-EXE: low-carbohydrate diet combined with exercise training (high-intensity interval training/moderate-intensity continuous training).
